# Determinants of the N content of *Quercus wutaishanica* leaves in the Loess Plateau: a structural equation modeling approach

**DOI:** 10.1038/srep26845

**Published:** 2016-05-27

**Authors:** Kaixiong Xing, Muyi Kang, Han Y. H. Chen, Mingfei Zhao, Yuhang Wang, Guoyi Wang, Chen Chen, Yang Liu, Xiaobin Dong

**Affiliations:** 1 State Key Laboratory of Earth Surface Processes and Resource Ecology, Beijing Normal University, Beijing 100875, China; 2 College of Resources Science & Technology, Beijing Normal University, Beijing 100875, China; 3 Faculty of Natural Resources Management, Lakehead University, 955 Oliver Road, Thunder Bay, ON P7B 5E1, Canada; 4 College of Life Sciences, Beijing Normal University, Beijing 100875, China; 5 Human Resource Development Center, Ministry of Land and Resources, Beijing 100812, China

## Abstract

Most terrestrial ecosystems are nitrogen (N)-limited. The elucidation of the multivariate relationships among environmental drivers, leaf morphological traits, and foliar N of dominant species which are critical to the functioning of forests remains a critical challenge for ecologists. We sampled leaves of *Quercus wutaishanica* across a broad natural gradient in the Loess Plateau, China, and employed structural equation modelling to evaluate the causal pathways and the relative importance of drivers of the foliar N per unit area (N_area_) and per unit mass (N_mass_). We found that (1) N_mass_ and N_area_ were primarily affected by leaf morphological traits instead of environmental variables and that leaf morphological traits accounted for most of their variations; (2) the total soil potassium and phosphorus and mean annual precipitation had different effects on N_mass_ and N_area_ via different pathways and path coefficients, whereas the mean annual temperature and total soil N had non-significant effects on N_mass_ and N_area_. Our results demonstrated that variations in N_mass_ and N_area_ within *Quercus wutaishanica* were strongly linked to their leaf morphological traits and that the leaf N was also influenced by mean annual precipitation and soil phosphorus and potassium instead of soil N in the Loess Plateau, China.

N comprises one of the most important limiting nutrients in plant growth and the net primary productivity of terrestrial ecosystems^1^,^2^. In forests, more than 40% of N of trees is stored within the leaves^3^ and foliar N exerts positive effects on their photosynthetic efficiencies and relative growth rates^4^,^5^,^6^,^7^. The N content within leaves in terrestrial ecosystems is intimately associated with environmental conditions and has been widely studied across species and at various scales^5^,^8^,^9^,^10^,^11^,^12^,^13^,^14^. Clarifying how the environment affects leaf N, which is an important predictor of light-use efficiency, is critical for predicting N status in terrestrial vegetation, especially in the context of temporal increases of N deposition over China^15^,^16^.

Plant trait variability due to both phenotypic plasticity and genetic diversity, which enables plant species to survive and reproduce under diverse environmental conditions, influences the response of species to environmental changes^17^,^18^. Fajardo and Piper^19^ firstly placed intraspecific variation of leaf mass per area (reciprocal of specific leaf area, SLA) and wood density of *Nothofagus pumilio* into the context of community ecology and assembly processes at a large scale, and found that intraspecific trait variation accounted for a large proportion of the total variation in traits. Subsequently, there is an explosion of studies on accounting for intraspecific trait variation, which may be critical for answering key questions and making predictions about plant community assembly and ecosystem functioning^20^,^21^. For example, it reported that strong but opposing responses among vs. within species for SLA and leaf N and phosphorus (P) concentrations, which are not typically accounted for in species-based measures of plant community^20^. With the accumulation of intraspecific trait variation researches, intraspecific trait variation accounted for 25% of the total trait variation within communities and 32% of the total trait variation among communities on average, which highlight global patterns in the relative importance of intraspecific trait variation in plant communities^21^.

Two indicators of leaf N content have been commonly used: mass-based (N_mass_) and area-based (N_area_) representations^9^,^18^,^22^,^23^,^24^. However, mass-based vs. area-based representations remain under discussion^1^,^7^,^25^. Wright *et al*.^1^ employed mass-based leaf traits to describe the universal leaf economic spectrum due to stronger correlations among the mass-based than the area-based leaf traits. Lloyd *et al*.^7^ noted that an area-based metric appears to be more logical because the primary function of leaves is to intercept light in the plant canopy; however, Westoby *et al*.^25^ noted that both representations warrant study according to different research purposes and needs.

Several environmental and leaf morphological variables, including specific leaf area (SLA), mean annual precipitation (MAP), mean annual temperature (MAT) and total soil nutrients, such as N, P and potassium (K), have been observed to affect N_mass_ and N_area_, which were primarily derived from correlative analyses^4^,^5^,^10^,^12^,^13^,^18^,^26^,^27^. Over expansive spatial scales, however, environmental drivers simultaneously influence N_mass_ and N_area_ as well as the species composition of the studied communities, making it difficult to separate the influences of environment from inherent differences between plant species. Therefore, environmental effects on the N content of leaves may be better understood within species^7^,^11^,^27^. Nevertheless, intraspecific variability, which plays in a critical role in community assembly processes and ecosystem functioning^20^,^21^, could result from both genetic variability and phenotypic plasticity^17^. Our understanding of the controls for the intraspecific variations of N_mass_ and N_area_ remains limited.

The morphological traits of leaves, such as specific leaf area (SLA), leaf dry weight (LDW) and leaf size (LS), may be associated with N_mass_ and N_area_. As a key morphological attribute, SLA is taken as the leaf-level cost of light interception^28^, which has been widely utilized as a key feature in studies of foliar N^1^ and plant growth strategies^29^. Additionally, LDW and LS may have independent effects on N_mass_ and N_area_. SLA is calculated by dividing LS by LDW, which may omit potentially independent effects of LS and LDW on foliar N. For example, the variation in leaf size is associated with major changes in within-leaf support investments and in large modifications in integrated leaf chemical (especially N concentration) and structural characteristics^30^, and there is also the decline of SLA along with the increase of LDW^31^. This suggests that N_mass_ and N_area_ may also be associated with LS and/or LDW in addition to SLA.

To better understand multivariate determinants of the leaf content of N within a species, we studied the N content of the leaves of the Liaotung oak (*Quercus wutaishanica*), a widely distributed dominant species of the deciduous broad-leaved forests, along natural gradients of climate and soil nutrient variability in the Loess Plateau, Northern China^32^. We examined the influences of MAT, MAP, total soil N (TSN), total soil P (TSP), total soil K (TSK), SLA, LDW, and LS on N_mass_ and N_area_ using two structural equation models (Fig. 1). Specifically, we hypothesized that soil nutrient contents have positive effects on N_mass_ and N_area_ and that increasing precipitation may reduce leaf N and soil nutrients due to increasing soil and leaf nutrient leaching^5^,^26^,^33^,^34^. We hypothesize that leaf SLA, LDW, and LS have profound influences on N_mass_ and N_area_ but that their directions of influences, i.e., positive or negative, are dependent on individual traits^4^,^5^,^10^,^12^,^13^,^18^,^26^,^27^. To test these hypotheses, we collected *Quercus wutaishanica* foliar samples across a wide range of environmental conditions in the Loess Plateau of northern China (Fig. 2, Table 1). Understanding the relative importance of these diverse pathways should help predict how N_mass_ and N_area_ respond to variations in climate, soil nutrients and the morphological traits of leaves.

## Results

### Correlation analysis

For the wide range of environmental variations we sampled, the mean N_mass_ and N_area_ were 23.59 mg·g^−1^ and 1.79 g·m^−2^, respectively (Table 1). The coefficient of variation (CV) for N_mass_ (15%) was lower than that for N_area_ (22%) (Table 1). Correlations of N_mass_ and other variables differed from those of N_area_ (Table 2). N_mass_ was positively correlated with SLA (*P* < 0.001) and LS (*P* < 0.001) (Table 2). N_area_ was negatively correlated with SLA (*P* < 0.001) and positively with LDW (*P* < 0.001) (Table 2). N_area_ increased with TSK (*P* < 0.001) and TSP (*P* = 0.023) (Table 2). There were significant correlations among SLA, LS, and LDW (Table 2). TSK and TSP were positively correlated (*P* < 0.001); however, neither had a significant correlation with TSN. MAT and MAP had a significantly negative correlation (*P* = 0.004) (Table 2).

### Model for N_mass_

**Figure 1:**
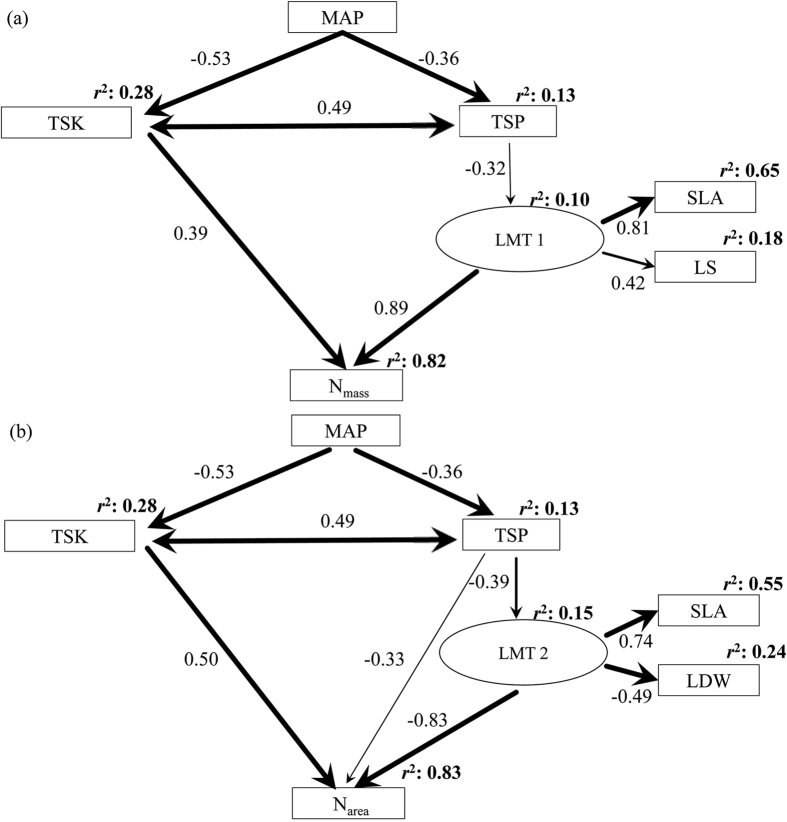
Results of the drivers for leaf nitrogen content. (**a**) Multiple drivers for leaf nitrogen concentration per mass (N_mass_). (**b**) Multiple drivers for leaf nitrogen concentration per area (N_area_). Single headed arrows indicate a hypothesized causal effect of one variable upon another. Double headed arrows indicate correlations. Insignificant (*p* > 0.05) paths were eliminated. Narrow arrows indicate *p* < 0.05; wider arrows indicate *p* < 0.01; and the widest arrows indicate *p* < 0.001. Signs on arrows indicate standardized regression weights or correlation indices. Signs at the top-right corner of each variable are the proportion of variance explained. LMT 1, leaf morphological traits incorporated from specific leaf area and leaf size; LMT 2, leaf morphological traits incorporated from specific leaf area and leaf dry weight; other abbreviations with units are explained in Table 1.

The model for N_mass_ (Fig. 1a) was a good fit with the data (Table 2), and environmental variables and leaf morphological traits (LMT 1, incorporating SLA and LS) accounted for 82% of the variation in N_mass_ (Tables 3 and 4, Fig. 1a). MAP had negative effects on both TSK and TSP, and TSK and TSP were positively correlated (Table 4, Fig. 1a). N_mass_ increased with TSK, whereas TSP had no direct effect on N_mass_ but had an indirect negative effect on N_mass_ through LMT 1 (Table 4, Fig. 1a). LMT 1 had a direct positive effect on N_mass_ (Table 4, Fig. 1a). MAT, TSN, and LDW were not included in the model because they had neither a significant direct or indirect effect on N_mass_ (Fig. 1a).

### Model for N_area_

The model for N_area_ (Fig. 1b) was also a good fit with the data (Table 3), and environmental variables and leaf morphological traits (LMT 2, incorporating SLA and LDW) accounted for 83% of the variation in N_area_ (Tables 4 and 5, Fig. 1b). The relationships between MAP, TSK and TSP were the same as those in the model for N_mass_. TSK also had direct positive effect on N_area_, which was higher than that on N_mass_. TSP had a direct negative effect and a positive indirect effect through LMT 2 on N_area_ (Table 4, Fig. 1b). LMT 2 had a negative effect on N_area_. MAT, TSN and LS were not included in the N_area_ model because neither had a significant direct or indirect effect on N_area_ (Fig. 1b).

### Partitioning of the explained variation of N_mass_ and N_area_

MAP explained 1% of the variation in N_mass_ and 7% of the variation in N_area_, and soil nutrients, which included TSK and TSP, explained 9% of the variation in N_mass_ and 18% of the variation in N_area_ (Table 5). LMT 1 explained 71% of the variation in N_mass_, and LMT 2 explained 59% of the variation in N_area_ (Table 5). MAP, TSK, and TSP explained 24% of the variation in N_area_, which was more than twice the variation explained in N_mass_ (10%) (Table 5).

## Discussion

The relationship between leaf morphological traits and leaf N content should be emphasized. Our results revealed multiple determinants for the N content of leaves (N_mass_ and N_area_) of *Quercus wutaishanica* in North China. In contrast with previous intraspecific studies that focused only on environmental influences^22^,^23^,^24^, we found that the variations in N_mass_ and N_area_ were more strongly associated with the morphological traits of leaves than environmental changes in climate and soil characteristics, with the latter including both direct effects and indirect effects via their influences on leaf trait variables, across the study area. This finding suggests that native ranges of morphological traits^18^,^20^,^21^ may be a strong determinant for foliar N of *Q. wutaishanica*. Future efforts, transplant studies for example^26^,^35^, are necessary to examine the genetic evidence associated with the foliar N, but study the variations and determinants across the native ranges of individuals (which reflects both plasticity and differences in genotype^11^) is an important first step.

SLA was strongly correlated with N_mass_ (*R* = 0.72) and N_area_ (*R* = −0.62) (Fig. 1). This finding was consistent with previous conclusions of interspecific comparisons^1^,^4^,^7^,^29^. LS included in SEM for N_mass_ (Fig. 1a) and LDW included in SEM for N_area_ (Fig. 1b) were reported here, however the important portion of variation in LS and LDW caused by genetically differences existing extensively across species^11^,^18^ lead to less attention than SLA. The negative correlation between SLA and LDW (Fig. 1b) was due to increased requirement for costly material support for a given leaf area with increasing LS^31^; however, there is no similar report for the relationship between LS and SLA. N_mass_ and LS were positively correlated in our SEM (Fig. 1a), which mean larger LS corresponding higher SLA. One of the major mechanisms by which plants adjust to resource imbalance is by allocating new biomass to the organs that acquire the most strongly limiting resources^36^. Larger leaves intercept more sunlight while cost more investment (positive correlation between LS and LDW, Table 1). Based on the extra mechanically support from twig^37^, the relative thinner or lower tissue density for the reduction of the burden for support tissue is achievable. Furthermore, compared to shrubs, larger leaves of trees are less disturbed by herbivores, which may lead to their less investment in defensive tissues. Hence the slower pace of LDW increase than LS lead to the higher SLA for larger leaves (Fig. 1a).

Both N_mass_ and N_area_ were affected by the total P and K in soils but not by the total N in soils. This finding is in contrast to the pattern reported in N addition experiments. Many planting experiments have reported that N addition in appropriate quantities may increase N_mass_ and N_area_^6^. However, chronic N addition in mature sugar maple forests initially increased N_mass_ and N_area_; notably, both indices began to decrease in the later stages of the experiment, which resulted in an insignificant effect of NO_3_^-^ addition on N_mass_ or N_area_^38^. Adult trees used in experiments have a far greater proportion of biomass than seedlings or young individuals for the storage nutrients, which leads to a considerably delayed response to environmental change and less dependence on environmental nutrient supplies through nutrient storage and resorption^14^,^39^. What’s more, our study species showed an N:P ratio of 21.78 (standard error = 0.54) in *Q. wutaishanica* (Table 1), indicating a relative N-surplus and P-limited environment^40^. There is most likely a correlation between the natural N supply and the level of N deposition today^41^. The annual bulk N deposition was 22–38 kilograms of N per hectare per year in the Loess Plateau in 2013^16^. With the enhanced N deposition over China around the year 2000 across China, there are significant increase of plant foliar N concentrations in natural and semi-natural ecosystems but no apparent soil N and P change^15^. The widespread increase in plant foliar N concentrations was caused by the cumulative effects of enhanced N deposition rather than alterations in soil^15^. Relative surplus foliar N accumulation from atmospheric depositions in our study area^15^ may lead to less dependency on soil N supply, which show us non-significant correlation between foliar N and the soil resident N variations. Alternatively, although soil total N can be strongly related to available N to plants including our study tree species, available soil N may be better linked foliar N. Future work could test the strength of relationships between soil total N vs soil available N to foliar N in the environment under high atmospheric N deposition.

Soil K was shown to have direct positive effects on both N_mass_ and N_area_, which has not been a focus in previous foliar N studies. K is a key element as an activator of the many enzymes that are essential for photosynthesis and respiration and as a contributor to the osmotic potential of cells^42^. A shortage of K leads to a decrease in the chlorophyll content of leaves in addition to a weakened capacity for photosynthesis^43^. Because the N within leaves is widely distributed in chlorophyll^44^, it has a direct positive effect on K within leaves in terms of both N_mass_ and N_area_^10^. The loss of K from leaves through leaching is higher than for other elements; thus, additional soil resident K ensures a supply of K to leaves^45^,^46^. Furthermore, K promotes the growth and secretion of ectotrophic mycorrhiza^47^, which exudes chemical compounds and enzymes into the rhizosphere and enhances the uptake of N^48^. At last, supplementary N could be provided to trees in high soil K environments due to greater net N mineralization and nitrification^12^.

We found that P in soils imparted a direct negative effect on N_area_, an indirect negative effect on N_mass_ and an indirect positive effect on N_area_ via leaf morphological traits. P is an essential element in photosynthesis that also improves the synthesis and transportation of photosynthetic products^43^. The addition of P may enhance the photosynthetic N use efficiency, leading to a negative effect on the distribution of N per unit area (N_area_ decreases)^49^. Comparing the positive effect of path “TSP → LMT 2 → N_area_” with the negative effect of path “TSP → N_area_”, the positive effect of TSP on N_area_ via LMT 2 complemented the decreased photosynthetic capacity per area caused by the negative direct impact of TSP on N_area_. Morphological traits of leaves could be one strategy of N utilization for optimizing N use efficiency and photosynthetic capacity of leaves. Additionally, considered relatively low level of TSP of the Loess Plateau region in China^50^, the photosynthetic products in low soil P habitats (with the exception of those that are conserved for leaf consumption) are distributed to meet increased needs for root growth^2^, leading to high N_mass_ and low leaf dry weight. However, specific leaf areas and leaf size increase to intercept additional light per unit mass and thus achieve higher use efficiency of leaf dry mass^28^. As a consequence, leaf dry mass per unit area and N_area_ are both decreased.

MAP had a negative total effect on the content of N in leaves, which was consistent with previous studies^8^,^26^. The MAP effects were indirectly accomplished through soil K and P, likely because higher MAP leads to extra soil nutrient leaching^5^,^33^. Foliar N may also be affected by increased leaf leaching^26^,^34^ and altered N absorption by the canopy associated with high MAP^46^. Our observed MAP effects on foliar N likely indicate that the leaf N leaching loss was counteracted by the increased foliar uptake associated with ongoing N deposition enhancement in China^15^.

Extensive comparisons between N_mass_ and N_area_ have been made. As previously noted, N_area_ should be selected for the leaf light intercept, whereas N_mass_ is allocated for resource distribution and plant growth^1^,^7^,^25^. In our SEMs, N_mass_ was also correlated with leaf size, whereas N_area_ was also correlated with the dry weight of leaves. This finding suggests that N_mass_ and N_area_ were both comprehensive functional leaf traits and that they should be simultaneously considered for the elucidation of foliar N traits. We also found a higher coefficient of variation (CV) in N_area_ (21%) than N_mass_ (14%), indicating that N_mass_ was a more stable variable than N_area_ in determining N content in leaves. This result is in agreement with a conclusion for 2548 species on a global scale^1^. Soil resident P and K and MAP had higher direct or indirect effects on N_area_ than N_mass_ and explained 24% of the variance in N_area_, which was more than twice that for N_mass_ (10%), indicating that N_area_ is a superior leaf N variable to reflect environmental variations.

## Conclusion

Our study established the determinants of intraspecific foliar N of natural forests over wide climate and soil nutrient gradients. To the best of our knowledge, this study represents the first attempt to quantitatively relate the N content of leaves to climate, soil nutrients, and the morphological traits of leaves at the intraspecific level. Our SEM demonstrated that N_mass_ and N_area_ of *Q. wutaishanica* were more strongly correlated to with morphological traits of leaves than to climate and soil nutrients and that different morphological traits of leaves were not equally correlated to N_mass_ and N_area_. Second, we found that soil resident K and P, but not N, exerted direct or indirect effects on N_mass_ and N_area_ of *Q. wutaishanica* trees. Thus, soil K and P are important and relevant nutrient variables for the management and conservation of *Q. wutaishanica* forests in the context of ongoing N deposition enhancement in China. Third, we found that both N_mass_ and N_area_ are critical indices for a full appreciation of foliar N within *Q. wutaishanica*. The lower CV of N_mass_ leads to a more stable indicator of the content of N in leaves than N_area_, whereas N_area_ is more sensitive to the variations in climate and soil nutrient conditions. Our findings highlight that foliar N content is influenced by multiple environmental drivers, whose relative importance can differ strongly depending on the different indices for foliar N content.

## Methods

### Study area

**Figure 2:**
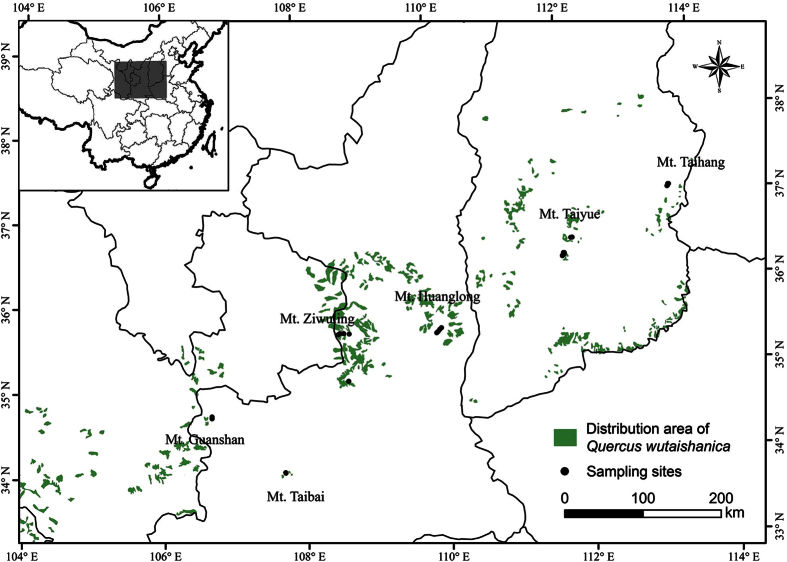
Locations of sampling sites. Black dots are location of sampling sites. Green-shaded portions are *Quercus wutaishanica* forest in the Loess Plateau. The map is made by ArcGIS 10.2 software, http://www.arcgis.com/features/.

The study was conducted in the Loess Plateau, Northern China, where *Q. wutaishanica* was primarily distributed across six mountains (34°03′–37°08′N; 106°41′–113°30′ E, Fig. 2). The mean annual temperature and precipitation in this region ranges from 4.1 to 10.3 °C and 554 to 880 mm, respectively, and the elevation ranges from 1252 to 2303 m (Table 1).

### Sampling strategy

To ensure a wide-ranging coverage of environmental conditions, we sampled *Q. wutaishanica* trees according to every 100-m elevation interval from each of the six mountains. Black dots show the locations of sampling sites. Green-shaded portions are *Quercus wutaishanica* forest in the Loess Plateau derived from the database for China’s terrestrial ecosystems (http://www.ecosystem.csdb.cn/ecosys/index.jsp) (Fig. 2). At each elevation sample site, we randomly sampled three dominant *Q. wutaishanica* trees, and within each sample tree, we collected 20 canopy leaves from the sunny side. The spatial location of each sample tree (for a total of 90 trees), latitude, longitude, and elevation were determined via GPS Garmin 60CSx (Garmin International Inc., Olathe, KS, USA) (Table 1). Surrounding the locations of each sample tree, within a 400 m^2^ proximity, three soil samples were randomly collected from a 0–20 cm soil depth, where the bulk of the fine roots of most plants occurs, and large amounts of N, P and K accumulate due to the uplift and releasing at the surface by plants through litter falls and fine root turnover^2^,^51^. Three soil samples were combined to a composite sample for chemical analysis in the laboratory.

### Functional traits

The leaf N_mass_, N_area_, N:P ratio, and morphological traits, i.e., SLA, LS, and LDW, were measured or calculated at the sample tree level. We first determined the average LS of each tree by scanning the fresh leaves. Subsequent to the oven drying of these leaf samples at 80 °C for 48 hours, we measured and calculated the average LDW. The dried samples from each tree were then pulverized using a plant sample mill and sieved through a 0.15-mm mesh screen. We employed an elemental analyser (Vario EL, Elementar Analyser systeme GmbH, Hanau, Germany) to determine the N concentration (N_mass_, mg/g) and P concentration^52^. N_area_ was calculated as N_mass_×LDW/LS. The N:P ratio was calculated as N_mass_/P_mass_.

### Environmental variables

Each soil sample was oven-dried at 105 °C for 24 hours and then pulverized using a soil sample mill. followed by sieving through a 0.15-mm mesh sieve and analysis for TSN using an elemental analyser (Vario EL, Elementar Analyser systeme GmbH, Hanau, Germany), TSP and TSK were performed using inductively coupled plasma atomic emission spectrometry measurements (ICP-AES, SPECTRO ARCOS EOP, SPECTRO, Germany). The MAP and MAT for each corresponding sample tree were derived from http://www.worldclim.org/ based on their spatial coordinates (latitude, longitude, and elevation).

### Statistical analysis

**Figure 3:**
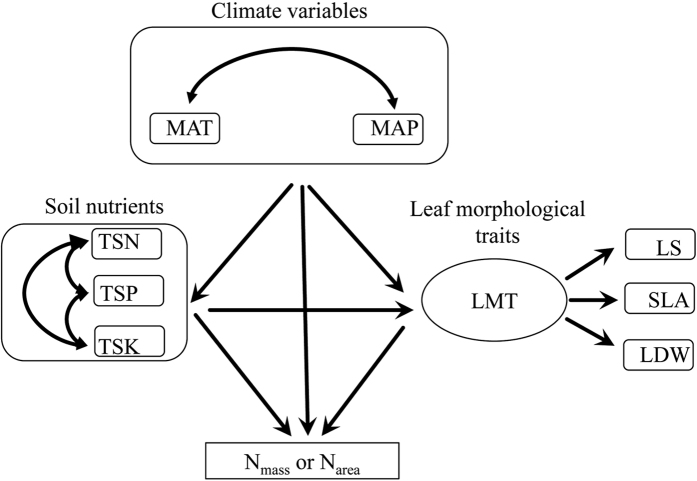
Illustration of all potential interaction pathways for leaf nitrogen in the study system. MAT, mean annual temperature; MAP, mean annual precipitation; TSN, total soil nitrogen; TSK, total soil potassium; TSP, total soil phosphorus; SLA, specific leaf area; LS, leaf size; LDW, leaf dry weight; LMT, leaf morphological traits; N_mass_, leaf nitrogen content per unit mass; N_area_, leaf nitrogen content per unit area.

The structural equation model (SEM) is an advanced and robust multivariate statistical method that enables hypothesis testing of complex path-relation networks^53^,^54^. SEM has increasingly been used in ecology to separate direct and indirect effects between exogenous and endogenous variables^55^. We first examined the bivariate relationships between several key hypothesized causal paths according to previous studies of the relationships between foliar N and driving variables. We then established a prior model based on the known theoretical construct including the key variables and their paths (Fig. 3). Three closely correlated leaf morphological traits (SLA, LDW, and LS, Table 1) were incorporated into a latent variable. Three observable soil nutrient variables (TSN, TSP, and TSK) were disposed as observable variables because these elements (N, P, and K) had different effects on foliar N caused by their different roles in plant physiology and growth^3^,^42^. Two observable climate variables (MAT and MAP) were also established as observable variables for the same reason. We subsequently used stepwise procedures that were guided by Akaike information criterion (AIC) values to obtain the most parsimonious set of predictors^54^. We adopted several indices to evaluate the suitability of the model: the chi-square test (χ^2^), the root square mean error of approximation (RMSEA), the adjusted goodness of fit index (AGFI), and the comparative fit index (CFI)^56^. Finally, we partitioned the explained variation (*R*^2^) of each variable to establish the influence of other predictors^57^.

## Additional Information

**How to cite this article**: Xing, K. *et al*. Determinants of the N content of *Quercus wutaishanica* leaves in the Loess Plateau: a structural equation modeling approach. *Sci. Rep.* **6**, 26845; doi: 10.1038/srep26845 (2016).

## Figures and Tables

**Table 1:** Main attributes of leaf traits (90 individuals) and environmental variables.

Variables	Mean	SE	Minimum	Maximum	CV (%)
Elevation (m)	1700	36.63	1252	2303	–
Longitude (°)	–	–	106.68233	113.50182	–
Latitude (°)	–	–	34.04959	37.13302	–
MAT (°C)	6.67	0.22	4.10	10.30	27
MAP (mm)	636.80	11.29	554.00	889.00	15
TSN (mg·g^−1^)	2.48	0.22	0.90	9.60	71
TSK (mg·g^−1^)	19.57	0.31	14.10	25.90	13
TSP (mg·g^−1^)	0.52	0.02	0.20	1.30	39
SLA (cm2·g^−1^)	13.81	0.43	7.50	28.40	27
LS (cm2·leaf^−1^)	35.82	1.35	19.50	72.40	28
LDW (g·leaf^−1^)	2.70	0.20	2.70	10.20	30
N_mass_ (mg·g^−1^)	23.59	0.41	17.60	33.70	15
N_area_ (g·m^−2^)	1.79	0.05	1.00	3.10	22
Leaf N:P ratio	21.78	0.54	13.15	41.76	21

Abbreviations: mean annual temperature, MAT; mean annual precipitation, MAP; total soil nitrogen, TSN; total soil potassium, TSK; total soil phosphorus, TSP; specific leaf area, SLA; leaf size, LS; leaf dry weight, LDW; leaf nitrogen per unit mass, N_mass_; leaf nitrogen per unit area, N_area_; standard error, SE; the coefficient of variation, CV.

**Table 2:** Pearson correlation coefficients between variables.

	N_mass_	N_area_	SLA	LS	LDW	TSN	TSK	TSP	MAT
N_area_	0.064								
SLA	0.63^***^	−0.63^***^							
LS	0.39^***^	−0.17	0.35^**^						
LDW	−0.03	0.45^***^	−0.37^**^	0.69^***^					
TSN	−0.23	0.05	−0.19	−0.07	0.09				
TSK	0.18	0.55^***^	−0.24^*^	0.03	0.28^*^	−0.08			
TSP	−0.07	0.27^*^	−0.27^*^	−0.01	0.24	0.03	0.58^***^		
MAT	−0.17	−0.04	−0.14	−0.11	−0.06	−0.07	0.09	0.13	
MAP	−0.21	−0.17	−0.01	−0.03	−0.02	0.59^***^	−0.53^***^	−0.36^**^	−0.34^**^

Significant effects are at *P* < 0.05 (*), <0.01 (**) and <0.001 (***).

Abbreviations are explained in Table 1.

**Table 3:** Structural equation model fit indices and evaluation criteria.

Index	evaluation standard or critical value for fit	Model for N_mass_	Model for N_area_
*χ*^2^		5.788	*χ*^2^ = 6.186
*p*	>0.05	0.565	0.403
AGFI	>0.90	0.923	0.903
RMSEA	<0.08	<0.001	0.021
CFI	>0.90	1.000	0.998

Abbreviations are: *χ*^2^, the chi-square test; RMSEA, the root square mean error of approximation; AGFI, adjusted goodness of fit index; CFI, the comparative fit index; N_mass_, foliar N per unit mass; N_area_, foliar N per unit area.

**Table 4:** Direct, indirect and total standardized effects on N_mass_ and N_area_ based on structural equation models (SEMs).

SEM model	Predictor	Pathway to foliar N	Effect
Model for N_mass_, Fig. 1a	Mean annual precipitation(MAP)	Total	−0.103
		Direct	–
		Indirect	−0.103^*^
	Total soil potassium (TSK)	Total	0.392
		Direct	0.392^***^
		Indirect	–
	Total soil phosphorus (TSP)	Total	−0.289
		Direct	–
		Indirect through LMT 1	−0.289^*^
	Leaf morphological traits incorporated from specific leaf area and leaf size (LMT 1)	Total	0.891^***^
		Direct	0.891^***^
		Indirect	–
Model for N_area_, Fig. 1b	Mean annual precipitation(MAP)	Total	−0.259
		Direct	–
		Indirect	−0.259^*^
	Total soil potassium (TSK)	Total	0.495
		Direct	0.495^***^
		Indirect	–
	Total soil phosphorus (TSP)	Total	−0.005
		Direct	−0.326^*^
		Indirect through LMT 2	0.321^*^
	Leaf morphological traits incorporated from specific leaf area and leaf dry weight (LMT 2)	Total	−0.833^***^
		Direct	−0.833^***^
		Indirect	–

Significant effects are at *P* < 0.05 (*), <0.01 (**) and <0.001 (***).

**Table 5:** Partitioning of explained variations of each variable.

Response variable	Variation explained by predictors				Total explained variation (%)
	MAP	TSK and TSP	LMT 1	LMT 2	
TSK	27.8				27.8
TSP	12.8				12.8
LMT 1	1.3	9.2			10.5
LMT 2	1.9	12.9			14.8
N_mass_	1.1	9.2	71.4		81.7
N_area_	6.7	17.7		58.9	83.3

Abbreviations are explained in Table 1.
